# Alveolar Ridge Preservation Using a Mixture of Alb-PRF and Alloplastic Bone Graft: A Case Report

**DOI:** 10.1155/crid/3007346

**Published:** 2025-03-13

**Authors:** Zein Moualla, Tarek Qasem, Ahmad Alnada

**Affiliations:** Department of Periodontology, Faculty of Dentistry, Damascus University, Damascus, Syria

**Keywords:** Alb-PRF, alloplast bone graft, alveolar ridge alteration, dentistry, oral surgery, radiological, sticky bone

## Abstract

Bone resorption occurring in the alveolar bone after tooth extraction constitutes one of the most fundamental problems that constitute a cosmetic and functional obstacle to treatment with dental implants or fixed prostheses at the site of extraction. A regenerative injectable complex, albumin and platelet-rich fibrin (Alb-PRF), composed of autologous albumin gel and platelet-rich fibrin (PRF) concentrate, it represents a new strategy that combines the advantages of PRF and albumin gel with slow absorption properties. The current case report included the extraction of a single-rooted tooth that was extracted atraumatically. Alb-PRF mixed with alloplast bone graft was used and applied within the socket. The socket was then covered with a piece of gelfoam. The case was followed up for 6 months. Radiographic and clinical measurements were performed, which showed the amount of alteration in both the bone and soft tissue after the extraction. Within the limitations of this case report, we concluded that alveolar ridge preservation using Alb-PRF mixed with alloplast bone graft has good results.

## 1. Introduction

Bone resorption occurring in the alveolar bone after tooth extraction constitutes one of the most fundamental problems that form cosmetic and functional problems to treatment with dental implants or fixed prostheses. In addition, this bone resorption may require additional procedures such as guided bone regeneration (GBR), which increases the complexity of the surgical procedure and treatment costs [1, 2].

The alveolar ridge preservation (ARP) aims to reduce resorption following tooth extraction by applying several biomaterials (such as xenografts bone or allograft bone and barrier membranes) during extraction [3]. This procedure stimulates and directs bone regeneration and reduces resorption following tooth extraction depending on the properties of the biomaterials as they are inductive or conductive [4].

Bone grafts have been used in bone regeneration and in ARP, and the highest percentage of use is for freeze-dried bone allograft, followed, in second place, by deproteinized bovine bone material (DBBM). It has been shown that the application of bone grafts in the ARP reduces postextraction resorption to clinically acceptable rates [5].

Platelet-rich fibrin (PRF) was previously used in GBR technology, but due to its rapid absorption, which resorbs completely within 10–14 days, a new technology was developed to obtain a material capable of staying for a longer period [6]. A complex of albumin and platelet-rich fibrin (Alb-PRF) represents a new strategy that combines the advantages of PRF and albumin gel with slow absorption properties that extend over 4–6 months [7].

## 2. Case Presentation

### 2.1. Diagnosis and Etiology

**Figure 1:**
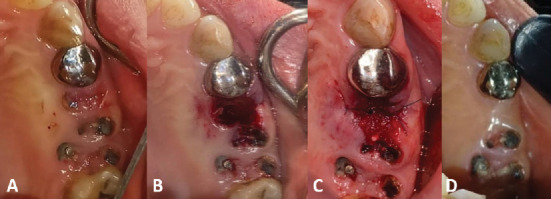
The surgical procedures: (A) before extraction, (B) after socket debridement, (C) after the socket sealed, and (D) follow up after 6 months.

A 42-year-old female had a nonrestorable right upper second premolar extraction, as shown in Figure 1A. She is a nonsmoker, does not suffer from any systemic diseases, and has no previous medical history of periodontal disease.

The patient participating in this study was informed of the details of the surgical procedure, the risks associated with it, and the alternative treatments. All her inquiries were answered, and she signed the informed consent through which she agreed to enter the study and to the use of images of the surgical procedure in scientific articles.

### 2.2. Treatment Objectives

The treatment aims to extract the second premolar and apply a mixture of alloplastic bone grafts with Alb-PRF to preserve the bone from resorption and prepare the tooth site for late implantation.

### 2.3. Treatment Alternatives

Alternative treatments for this case are spontaneous healing of the extraction socket or immediate implantation in the extraction site. It may be decided to apply this technique (ARP) after obtaining informed consent from the patient for the procedure.

The emergency in the event of failure previously decided in this case in the event of postextraction alveolitis or alveolar infection is to remove the bone graft from the socket and repeatedly wash the socket until complete healing occurs.

### 2.4. Treatment Progress

Infiltration anesthesia was performed using lidocaine 2% with epinephrine 1:80,000 (ESEP, 3M, Los Angles, United States) in the buccal and palatal area adjacent to the tooth to be extracted. An atraumatic extraction was performed using a Periotomes (JKSurgical, Kabul, Pakistan) to avoid breaking the buccal bone plate of the extraction socket. After completing the extraction, the integrity of the socket walls was confirmed using a periodontal probe (UNC-15; JKSurgical, Kabul, Pakistan). It was found that the extraction socket was of Type I according to Elian 2007 Classification [8]. Then, the socket was debrided using a bone curette to remove the remaining periodontal ligament and the granulation tissue; the socket was washed with saline Figure 1B.

**Figure 2:**
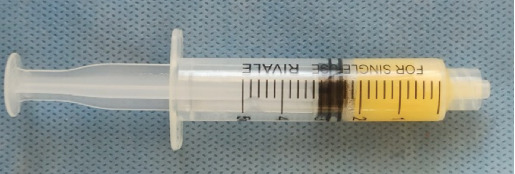
Albumin after protein reconstruction.

**Figure 3:**
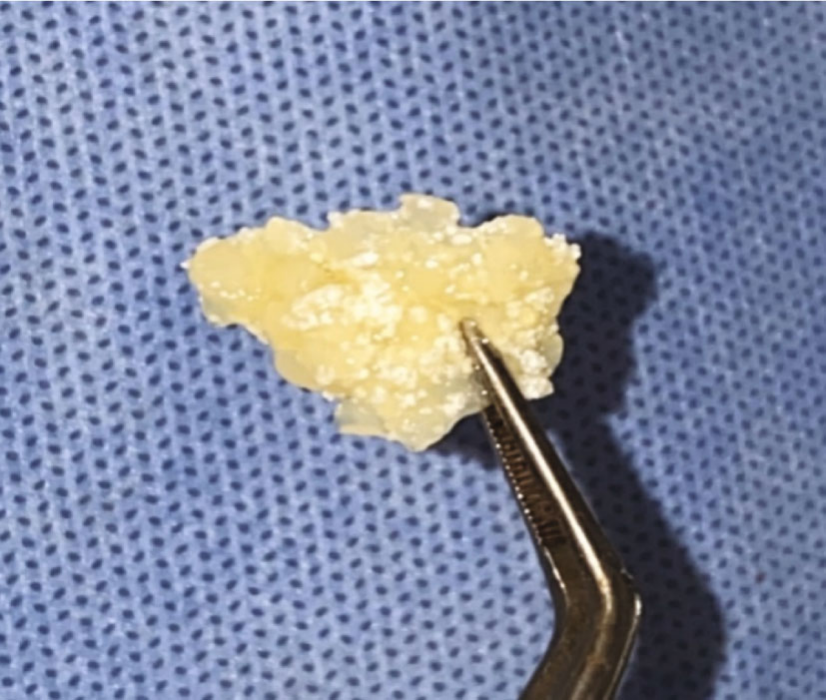
The mixture of Alb-PRF and alloplast bone graft.

Then, 10 mL of blood is drawn from the patient, placed in a plastic tube and centrifuged with 700*g* for 8 min using a centrifuge (Bio-PRF, Florida, United States) [7]. Platelet-poor plasma which does not contain cells and contains 60% albumin protein was drawn with a plastic sterilized syringe and placed in an air convection oven (BioHeat; Bio-PRF, Florida, United States) at a temperature of 75° for 10 min (Figure 2). The proteins restructure themselves into a denser organized structure called an albumin gel. Albumin gel is mixed with platelet-rich plasma and alloplastic bone graft (Medtronic, Dublin, Ireland) which contain 40% tricalcium phosphate (TCP) and 60% hydroxyapatite (HA). The mixture (Figure 3) was applied to the extracted socket [9].

After compacting the mixture into the socket, we cut a suitable piece of gelfoam, sealed the extraction socket with it, and sutured using 5/0 nylon thread (VertMed, Syke, Germany) Figure 1C.

### 2.5. Treatment Results

We performed a CBCT radiograph immediately after the surgical procedures to compare the dimensions 6 months after the extraction.

**Figure 4:**
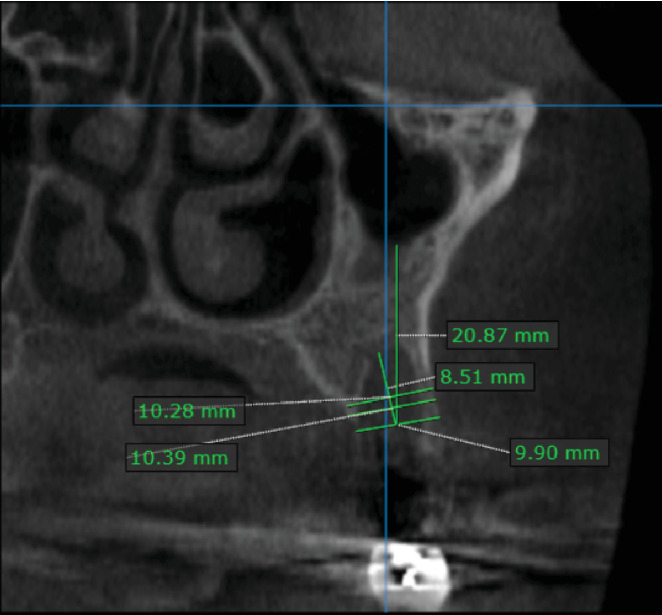
The radiographic measurements immediately after the surgical procedures.

Radiographic measurements were made on the image taken immediately after surgery using OnDemand3D software v9.0 (Cybermed Inc., California, United States), and it was found that the height of the socket was 8.51 mm, and the width of the socket at the level of 3 mm from the socket was 10.39 mm, and at the width at level of 5 mm, it was 10.28 mm Figure 4.

The surgical sutures were removed after a period of 14 days, and the healing was very good according to Landry's index for soft tissue healing, and reached excellent in the fourth week of extraction, and still stable for 6 months (Figure 1D).

**Figure 5:**
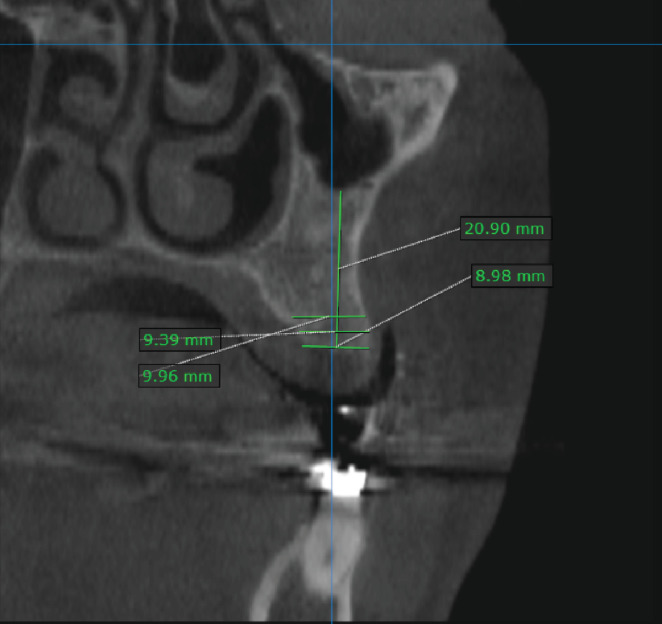
The radiographic measurements after 6 months of the surgical procedures.

The healing was reevaluated radiographically using a CBCT image taken 6 months after the extraction (Table 1), and it was found that the alveolar height had not decreased (given the line connecting the middle of the alveolar orifice and the floor of the maxillary sinus as a reference point for comparison). The width of the socket at the level of 3 mm was 9.39 mm, and at the width at level of 5 mm, it was 9.96 mm, and the radiographic bone density in it was 9.96 mm (Figure 5).

## 3. Discussion

The traditional treatment for unrestorable teeth is traditional extraction and spontaneous healing. Still, this method leads to bone resorption that may prevent us from performing dental implants or tooth replacement [10].

Many studies have been conducted to reduce the subsequent bone resorption and applied bone grafts [11], membranes [12], PRF [13], and socket seal surgery [14]. Still, until now, no ideal treatment prevents bone resorption following extraction.

Alb-PRF was used because of its relatively slow resorption time which allows bone formation compared to L-PRF which is absorbed within 7 days [7].

The extraction was performed atraumatically using periotomes and without flap, elevation to reduce the socket trauma, which leads to an increase in bone absorption [15]. The bone curettes were used to completely curettage the dental ligament to ensure that its cells are not inserted into the bone graft or the healing process following the extraction, and to help increase the blood flow and the migration of the bone cells from the bone marrow [16].

The vertical bone resorption is one of the most important problems facing tooth replacement with implants or fixed prosthesis due to the resorption in bone structures that offend the aesthetic and function of compensation [1, 17].

The absorption occurs in the buccal plate of the socket and may reach a complete absence of the buccal plate, especially if it is with a thickness of less than 1 mm, and thus causes horizontal loss along the extraction socket [1, 2].

Landry's index was used to measure healing in soft tissues because the index studies soft tissue healing with the second intention, which is similar to the healing in the socket after extraction [18].

Thakkar et al.'s [19] clinical study aimed to compare two techniques for socket preservation after teeth extraction, which included the application of allograft bone graft with or without I-PRF. The results of this study showed the mean of vertical bone changes in the group with I-PRF was −1.08 ± 0.43.

Clark et al.'s [20] clinical study assesses the effectiveness of PRF alone or with bone graft in the ARP after teeth extraction, the sample included 40 sites. The study showed that the sticky bone group had less horizontal bone changes (1.9 ± 1.1).

However, it could be interesting in the future to test also other adjunctive treatments such as ozone [21], photobiomodulation [22], and paraprobiotics [23] in order to understand their potential effect on tissue healing.

One of the limitations of this study is that it lacks the histological part that was scheduled in the implant session, and it also lacks the data of the initial insertion torque and the implant stability quotient.

## 4. Conclusion

The problem of bone loss after extraction is one of the problems that negatively affect the replacement of these teeth with fixed prostheses or the use of dental implants. The rapid absorption of PRF, which is 14 days, has limited its use with bone grafting, especially with the sticky bone. Recently, the bone graft mixture with Alb-PRF has been used, which has a slow absorption sufficient to achieve bone formation. Within the limitations of this case report, we concluded that ARP using Alb-PRF mixed with alloplast bone graft has good results.

## Figures and Tables

**Table 1:** The timeline of the treatment and the surgical procedure.

**Date**	**Procedure**
5/11/2023	Patient's first visit, diagnosis, and treatment plan
19/11/2023	Surgical procedure
26/11/2023	Recall CBCT scan 7 days after the procedure
3/12/2023	Suture removal 14 days after the procedure
17/12/2023	Recall soft tissue healing recording
26/5/2024	Recall CBCT scan 6 months later

## Data Availability

The authors can provide data on demand.
